# ARV regimen choice and co-morbidities

**DOI:** 10.1186/1471-2334-14-S2-S9

**Published:** 2014-05-23

**Authors:** Barry Peters

**Affiliations:** 1 Infectious Diseases Department, King’s College, London, SE1 1UL, United Kingdom

## 

The main HIV co-morbidities include coronary heart disease (CHD), metabolic syndrome and lipodystrophy, renal impairment, fragility fractures, neurocognitive disease, and the emerging complications of insulin resistance and fragility fractures. The evidence base for toxicities seen with ART is gained mainly from comparative efficacy studies of ART regimens, where toxicity is not the primary endpoint, or cohort studies, with their inherent limitations.

## ART choice to mitigate co-morbidities

### Coronary Heart Disease

Most HIV cohorts show an increase in CHD, thought due to a direct pro-inflammatory consequence of HIV infection and increased risk factors. Some drugs, notably the protease inhibitors, and to some extent others including efavirenz, increase lipids with a detrimental total cholesterol (TC) HDL ratio; by increasing HDL, neviripine improves the ratio. However, cardiac risk is more important in selection of drugs than lipid levels alone. The evidence of from DAD and other cohort for increased CHD with abacavir probably represents a channelling bias, and most other cohorts have not found this association. The integrase inhibitors, CCR5 inhibitors, eg maraviroc, and the newer PIs, including Atazanavir and Darunavir, have less lipid effects.

### Metabolic effects, including lipodystrophy, metabolic syndrome, IR

The cessation of thymidine analogue use has markedly reduced lipoatrophy and several other metabolic effects. Again the integrase inhibitors, maraviroc, and for insulin resistance the newer PIs, are “clean” in these respects.

### Renal impairment

Tenofovir is unlikely to be a problem if renal function, incl urine protein: creatinine ratio, is monitored.

### Fragility fractures

The marked increase in osteoporosis, and reduced bone mineral density (BMD) is likely to herald a commensurate rise in fragility fractures as our patients age and may justify avoiding drugs associated with accelerated BMD loss, the protease inhibitors and tenofovir, in the elderly and those with greatest fracture risk. Statins reduce CHD risk, may improve BMD, but are associated with increased IR and diabetes.

### Neurocognitive disease

As well as issues of tolerability, there is data suggesting that efavirenz might have long-term neurological effects.

## Conclusions

The wide and increasing choice of ART means that considerations of avoidance of short and long term co-morbidities can be fully addressed without compromising efficacy. Thymidine analogues are rarely justified. ART co-morbidities are best mitigated by screening for risk of co-morbidities, and use of use of those drugs that are least likely to exacerbate these risks.

The integrase inhibitors, CCR5 blockers, non-thymidine NRTIs, neviripine appear to be preferential to some other agents, but for good access, pricing and a tiered tariff for developing countries will be important determinants.

